# Editorial: The Role of Letter-Speech Sound Integration in Normal and Abnormal Reading Development

**DOI:** 10.3389/fpsyg.2020.01441

**Published:** 2020-07-07

**Authors:** Jurgen Tijms, Gorka Fraga-González, Iliana I. Karipidis, Silvia Brem

**Affiliations:** ^1^Department of Psychology, University of Amsterdam, Amsterdam, Netherlands; ^2^Department of Child and Adolescent Psychiatry and Psychotherapy, University Hospital of Psychiatry, University of Zurich, Zurich, Switzerland; ^3^Center for Interdisciplinary Brain Sciences Research, Department of Psychiatry and Behavioral Sciences, Stanford Medicine, Stanford University, Stanford, CA, United States

**Keywords:** reading development, dyslexia, letter-speech sound integration, reading disabilities, neural circuits

Learning to read is a central focus of education as it enables us to successfully participate in society and develop as individuals. In this process, a crucial milestone toward expert reading is the ability to read fluently, that is, to quickly and effortlessly access the meaning of print. However, this is not an innate skill and has to be learned at school through explicit instruction and extensive practice. Specific brain networks functionally specialize to serve reading-related cognitive processes. In particular, areas involved in visual recognition of symbols and auditory processing of language are at the heart of this adaptation. Their specialization is essential in the first stages of learning how to read. As a vital part of this process, the crossmodal (audiovisual) integration of letters and speech sounds constitutes the key starting point toward an effective brain network that enables fluent reading (Blomert, 2011).

Letter-speech sound (LSS) integration is an important process to be studied in order to better understand progress in typical reading acquisition and impairments in developmental dyslexia. A failure in the automation of LSS associations has been consistently reported in impaired readers of different orthographies (Richlan, 2014). In addition, optimizing this associative process seems to be a key ingredient for positive outcomes in training and intervention aimed at improving reading fluency (Mehringer et al., 2020; Patel et al.). From a neurodevelopmental perspective, neuroimaging studies suggest that the input from audiovisual integration areas into visual processing areas is a primary drive of visual specialization, crucial in the later stages of the reading network development (Brem et al., 2010; Fraga González et al., 2017; Pleisch et al., 2019).

Several important research questions remain to be clarified. First, what constitutes typical and atypical developmental pathways of LSS integration and how do these relate to successful and unsuccessful reading acquisition? Second, do more fundamental deficits such as deficits in general associative learning mechanisms obstruct LSS integration in impaired readers? Third, and more relevant to clinical practice, what is the optimal way to facilitate the process of LSS integration and automation? In the current Frontiers' Research Topic we present a collection of original research articles from different disciplines that are, directly or indirectly, advancing our knowledge on these questions. We divided the papers in this Research Topic conceptually into three sections: (1) cognitive and linguistic processes related to the development of letter-speech integration, (2) a neurocognitive window into letter-speech sound binding, and (3) interventions to support reading development.

## Cognitive and Linguistic Processes Related to Letter-Speech Sound Integration

This first section is dedicated to mechanisms related to the associative learning processes in LSS integration. The studies in this section help us specify cognitive abilities and perceptual processes important for reading acquisition, as well as deficits in specific learning disorders, including dyslexia and dysgraphia. Experimental manipulation of the visual and auditory properties of LSS offer valuable insights to the fundamental mechanisms of these cognitive and linguistic processes. In addition, relating performance in such experimental paradigms with literacy outcomes provides a better understanding of how specific aspects of cognition relate to typical and atypical literacy development. For instance, artificial script learning paradigms are excellent experimental tools to examine these issues and to find new predictors of reading abilities.

The first paper of this section (Pavlidou and Bogaerts) examined the role of implicit statistical learning (ISL) in reading acquisition. The results point to the relevance of perceptual modality in ISL, and show an interesting association between visual ISL and phonological awareness. Pavlidou and Bogaerts discuss how visual ISL could facilitate mapping letters and speech sounds in novice readers. Keetels et al. approached the atypical integration of letters and speech sounds in dyslexia by examining the ability to adjust one's perceptual interpretation of ambiguous speech input in accordance with contextual information. Results revealed that adults with dyslexia were, in contrast to typical readers, unable to use text to recalibrate their phoneme categories, whereas no deviations in their ability to recalibrate by lipread speech were found. This result supports a LSS integration deficit in dyslexia, but suggests this does not extend to a more general audio-visual integration deficit. Interestingly, Romanovska et al. using the same paradigm, failed to find a difference in recalibration by written text between young readers with and without dyslexia. The authors emphasize the importance of taking dynamic developmental processes into account, and specifically, they point to the potential role of changes in the temporal integration window for LSS coupling during development. From a linguistic perspective, Caccia et al. showed that in Italian, pitch is the most reliable acoustic cue in stress perception in words for adults and, less markedly so, for typical reading children. In contrast, Italian children with dyslexia did not seem to rely as much as typical readers on pitch for stress perception. Following their results, Caccia et al. discuss the relevance of language-specific features in studying atypical reading development.

Although previous research has shown many similarities between reading disabilities (dyslexia) and spelling disabilities (dysgraphia), there is a dearth of research specifically dedicated to dysgraphia. Döhla et al. therefore investigated which cognitive deficits are associated with spelling deficits in dysgraphia. A cluster analysis revealed that children with dysgraphia could be split into two distinct clusters, one with auditory deficits and the other with deficits in visual magnocellular functions. The authors discuss the implications of these findings for developing more individually tailored interventions.

The last two studies of this section used a paradigm of artificial LSS learning to mimic the initial stages of reading acquisition. Horbach et al. developed a paradigm in which preliterate children had to map morse-code symbols to speech sounds, and subsequently followed the children's reading development over a 3-year period. The performance on this learning paradigm turned out to be a particularly relevant predictor of reading fluency and reading comprehension skills 3 years later. Law et al. also examined artificial LSS learnability with Hebrew letters and speech sounds, comparing children with dyslexia and typical readers in grade 3. The results showed a reduced ability of children with dyslexia to use the newly learned LSS correspondence for reading words presented in the novel script. However, in contrast to Horbach et al. this study found no significant independent contribution of artificial LSS learning to reading skills.

## A Neurocognitive Window into Letter-Speech Sound Binding

The studies included in this section used a wide range of non-invasive neural imaging and modulation (or stimulation) methods to study letter-speech sound binding and how it relates to reading. These methods include event-related potentials (ERP) of electroencephalography, magnetocencephalography (MEG), functional magnetic resonance imaging (fMRI), transcranial direct current stimulation (tDCS), and magnetic resonance spectroscopy (MRS). Common to all studies is their special interest on measures of brain activation and connectivity in parieto-temporal brain regions, because of their important role in the processing of audiovisual information.

In the first paper of this section, Richlan et al. presents a review on the neural networks associated with LSS integration in typical and atypical reading development. The review suggests a putative neurocognitive deficit specific to the crossmodal integration of letters and speech sounds that hinders the emergence of a functional neural system for reading. In concordance with this suggestion, Plewko et al. show that alterations in brain activity during LSS association can be detected at very early stages of reading acquisition in kindergarten and first grade between children with and without familial risk for dyslexia. Their results suggest that an increased response of the left superior temporal cortex to incongruent LSS pairs reflects an early stage of automatization. Absence of such a distinct response to incongruent information during this early stage of reading acquisition is suggested to potentially cause reading problems through deficient suppression of irrelevant information. In an ERP study, Kemény et al. also demonstrate a deviation in neural responses in a stroop-like interference LSS integration task in children with combined reading and spelling deficits, compared to typically developing peers. Notably, ERPs of children with isolated deficits in spelling did not differ from those of typically developing children, suggesting that deficits in automatized LSS associations may be specifically associated with reading impairments.

Both Younger and Booth, and Xu et al. emphasize the role of parietotemporal regions in the early stages of learning to read. Using transcranial direct current stimulation (tDCS) to manipulate parietotemporal function, Younger and Booth revealed that stimulation of these brain areas can enhance the learning of new grapheme-phoneme associations. Interestingly, the results of Younger and Booth suggest that whereas parietotemporal function may be critical to new, initial learning, its role in continued reading development is likely to change afterwards. In line with these findings, the results of the MEG study of Xu et al. with Finnish school children emphasize the crucial role of the parieto-temporal cortex in the early phase of reading. They show that audiovisual integration effect of letters and speech sounds are most pronounced in parietotemporal regions and correlate with reading and writing skills.

Faísca et al. provide novel insights into processes involved in visual word recognition. In their ERP study, they show task dependent effects of lexicality on early ERPs (within 200 ms) in expert adults readers. This result suggests that visual word recognition is not simply the consequence of letter-speech integration only, but results from an interplay between various bottom-up and top-down processes.

The study of Smith et al. uses fMRI to examine developmental changes in functional connectivity in children's neural reading network over a 2.5-year period. This study provides evidence that improvements in reading skill over time are predicted by the nature and degree of changes among connectivity patterns within the reading network. More specifically, an overall increase in processing coherence among regions of the reading network, was shown to be a critical driver of growth in reading proficiency.

The final study in this section, by Del Tufo et al., examines the mediating role of crossmodal integration of visual and spoken word representations in the relationship between neurochemical concentrations and reading proficiency using MRS. The results revealed that the effect of cross-modal word matching mediated the relationship between increased glutamate (a suggested index of “neural noise” or random variability in neuronal firing) and poorer reading ability as well as the relation between increased choline and poorer reading ability. In addition, lower GABA and higher N-acetyl-aspartate (NAA) predicted faster cross-modal matching reaction times. These results are discussed vis-à-vis a biochemical framework in which the ability of neurochemistry to predict reading ability may at least partially be explained by cross-modal integration.

## Interventions to Support Reading Development

This section presents several intervention approaches for reading in different languages that aim at supporting struggling readers or facilitating typical reading development. In some of these interventions, LSS integration constitutes a central training component.

Patel et al. evaluated the effects of a game-based intervention, GraphoLearn, that trains LSS mappings in English reading skills of young struggling readers in India. In this small-scale study, a group of 7-year-old children was randomly allocated to either the GraphoLearn training or a control Math-game training. The results revealed that, compared to the control training, GraphoLearn led to significant improvements in children's letter-sound knowledge, a critical factor in early reading development.

The three other studies of this section did not focus on children with reading impairments but investigated the potential of specific intervention mechanisms to promote reading development. In the study of Pinto et al. a training called PASSI (Promoting the Achievement of Sound-Sign Integration) aimed to improve kindergarteners' conceptual knowledge of the Italian writing system. 159 Italian children (3–5 year old) were randomly assigned to either the experimental training or a control group. Results revealed positive effects of this type of training on several emergent literacy skills. Siu et al. were interested whether metalinguistic awareness (addressing phonetic-symbolic and semantic-symbolic mappings) or working memory training could be effective for Chinese reading skills. In this small-scale study, second graders in Hong Kong were randomly assigned to a metalinguistic training group, a working-memory training group, or a waitlist control group. The results of the study suggest that metalinguistic awareness training enhanced phonological skills, whereas the working memory training enhanced memory span, but both training groups improved similarly in word reading fluency in Chinese and English compared to the control group. In the last study, Su et al. compared effects of two reading styles, reading with a narrator vs. reading independently, on eye movements and early literacy skills of young children (4–6 years old). Children were randomly assigned to one of the two reading approaches, and effects of the narrator were evaluated by analysis of eye movement patterns. Based on differences in fixation patterns during text reading, Su et al. concluded that children in kindergarten profit from reading with a narrator because this supports the acquisition and consolidation of mappings between speech and text.

## Concluding Remarks

To sum up, we present a collection of studies from diverse fields that contribute to our understanding of a milestone in human cognition that is reading acquisition. The studies in the first section successfully pointed at associative learning mechanisms and markers that are important contributors to our capacity of integrating letters and speech sounds to become successful readers. The neuroimaging studies in section two advance our knowledge of typical and atypical development of brain networks that enable us fluent reading, clarifying the role of crossmodal integration regions as part of a connected reading network. Finally, the importance of LSS integration for reading finds support in the intervention studies in section three, which demonstrate its relevance to the design of support and remediation programs for reading across languages. Although there are still important gaps in our current models of typical and atypical reading abilities and reading acquisition, we are certain that the studies presented in this Frontiers Research Topic extend our knowledge and open new interesting venues for future research. The investigation of audiovisual integration of letters and speech sounds constitutes a fascinating and complex topic which encompasses interdisciplinary knowledge from both fundamental and applied research domains. We look forward to more exciting new findings and hope that the integrative efforts of so many international and multidisciplinary labs continue yielding progress in our understanding of human learning and cognition.

## Author Contributions

All authors listed have made a substantial, direct and intellectual contribution to the work, and approved it for publication.

## Conflict of Interest

The authors declare that the research was conducted in the absence of any commercial or financial relationships that could be construed as a potential conflict of interest.
